# Oral somatosensatory acuity is related to particle size perception in chocolate

**DOI:** 10.1038/s41598-019-43944-7

**Published:** 2019-05-15

**Authors:** Scott P. Breen, Nicole M. Etter, Gregory R. Ziegler, John E. Hayes

**Affiliations:** 1 Sensory Evaluation Center, The Pennsylvania State University, University Park, PA 16802 USA; 2 Department of Food Science, College of Agricultural Sciences, The Pennsylvania State University, University Park, PA 16802 USA; 3 Department of Communication Sciences and Disorders, College of Health and Human Development, The Pennsylvania State University, University Park, PA 16802 USA

**Keywords:** Human behaviour, Neurology

## Abstract

Texture affects liking or rejection of many foods for clinically relevant populations and the general public. Phenotypic differences in chemosensation are well documented and influence food choices, but oral touch perception is less understood. Here, we used chocolate as a model food to explore texture perception, specifically grittiness perception. In Experiment 1, the Just Noticeable Difference (JND) for particle size in melted chocolate was ~5 μm in a particle size range commonly found in commercial chocolates; as expected, the JND increased with particle size, with a Weber Fraction of ~0.17. In Experiment 2, individual differences in touch perception were explored: detection and discrimination thresholds for oral point pressure were determined with Von Frey Hairs. Discrimination thresholds varied across individuals, allowing us to separate participants into high and low sensitivity groups. Across all participants, two solid commercial chocolates (with particle sizes of 19 and 26 μm; i.e., just above the JND) were successfully discriminated in a forced-choice task. However, this was driven entirely by individuals with better oral acuity: 17 of 20 of more acute individuals correctly identified the grittier chocolate versus 12 of 24 less acute individuals. This suggests phenotypic differences in oral somatosensation can influence texture perception of foods.

## Introduction

Food texture is a perception^1^, arising from the interaction of a food with mechanoreceptors in the oral cavity; it depends not only on the physical structure of the stimulus, but also the neural impulses carried by multiple afferent nerves^2^. Despite being a key driver of the acceptance or rejection of foods^3^, oral texture perception remains poorly understood relative to taste and smell, two other sensory inputs critical for flavor perception. It has been argued that texture is not typically noticed when it is within an acceptable range, but that it is a major factor in rejection if an adverse texture is present^4^. For chocolate specifically, oral texture is a critical quality attribute, with grittiness often being used to differentiate premium chocolates from bulk chocolate.

Chocolate is a semi-solid suspension of fine particles from cocoa and sugar dispersed in a continuous fat phase, making it an ideal food for the study of texture. Of the three types of chocolate — dark, milk, and white — dark leads the global market in total sales^5^. Increased consumer interest and consumption of dark chocolate is presumably due to the putative health benefits and properties of dark chocolate that have been widely reported in mass media. For example, various reports suggest bioactive components in cocoa may have a beneficial effect on cognitive function and cognitive decline^6^, may help increase HDL cholesterol levels^7^ and may have anti-inflammatory activity^8^. By definition, dark chocolate does not contain dairy fat, making it a slightly simpler system than milk chocolate for the study of texture; given this, as well as its global commercial relevance, we used dark chocolate as our model system.

The chocolate making process requires numerous steps to convert cocoa beans and sugar into finished chocolate. In particular, the refining step is especially critical for the production of the desired smooth texture^9,10^. During refining, physical force is applied to the cocoa paste to break down the cocoa and sugar particles into smaller sizes. However, this step is also a common bottleneck in production, as finer particle sizes require more processing time and higher energy inputs^11^. Roll refining is the most common means to reduce particle sizes in commercial chocolate manufacturing^9^, although hammer or ball mills are also used. Because chocolate production is energy intensive, manufacturers wish to reduce refining time to minimize cost. However, such concerns need to be balanced against product acceptability.

**Figure 1:**
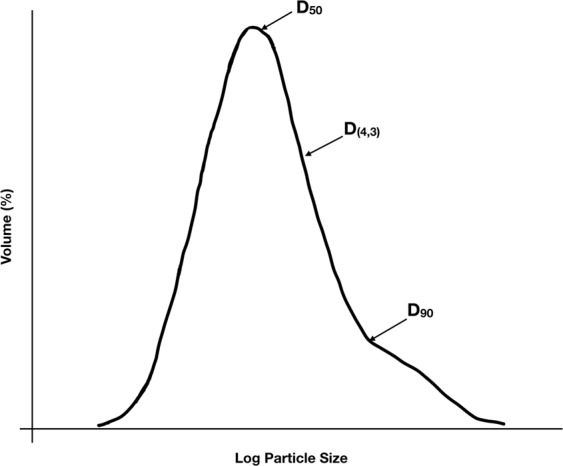
Stylized example of a particle size distribution obtained for chocolate using laser diffraction, with several commonly used summary parameters indicated.

In chocolate, the particle size distribution (PSD) is a fundamental characteristic which has a large influence on both rheology and texture^9–11^. Unsurprisingly, the largest particles have the most influence on mouthfeel and grittiness. Accordingly, one widely-used measure of particle size in chocolate manufacturing is the D_90_ value, which indicates the size of particles at the 90^th^ percentile of the distribution by volume – that is, 90% of the particles within the sample are finer than the D_90_ size. When measured via laser diffraction, this value correlates with the sensory perception of the largest particles^9^. Another widely used parameter is the D_(4,3)_ value, which refers to the volume or mass moment mean (i.e., the De Brouckere mean diameter). A stylized example of a particle size distribution found in chocolate is shown in Fig. 1. Here, different chocolates were quantified using the D_90_ and D_(4,3)_ values. It is widely claimed that chocolate is perceived as sandy or gritty when particles are larger than ~25–35 μm^11,12^. Critically however, we have been unable to identify the original source for this value. The closest we have been able to find is a 1967 report by Rostagno, who reported that chocolate was unacceptably coarse when 20% of the particles exceed 22 μm^13^.

When two products are perceived to have the same grittiness, the products may not be identical in physical composition and particle size distribution; rather, the difference in grittiness may be too small to be perceived as different. In psychophysics, the Just Noticeable Difference (JND) is defined as the smallest change that can be detected between two stimuli (e.g.^14^). Using JND values measured empirically for stimuli across a range of values, the Weber Fraction can then be calculated. The Weber Fraction (K) is an index of the sensitivity of a sensory system to detect changes in product attributes^15^. Despite the use of particle size as a control point in the chocolate industry (e.g.^16^), there is no work in the open literature using relevant psychophysical methods^17^ to quantify the JND for particle size in chocolate.

The mechanisms for perception of texture in the mouth remain relatively poorly understood relative to research on touch for other body parts like the hands^18–21^ and eyes^22^. Measurement of tactile point pressure sensitivity is widely employed in neurology and other disciplines as a measure of peripheral nerve function through the use of Von Frey Hairs (VFH), sometimes called Semmes-Weinstein monofilaments^23^. VFH are small calibrated monofilaments which deliver a specific amount of pressure. They are low cost, noninvasive, relatively easy to use and provide a fast way of estimating somatosensation^24^. VFH have been used to measure mechanical sensitivity in both infants, adults and even animals^25^ on the hands, feet (e.g.^26–28^) and face (e.g.^28^). Although prior work using VFH suggests pressure sensitivity on the tongue varies across people^29^, research on differences in tactile perception in the oral cavity are relatively limited^30^. Other previous studies have reported mechanical detection thresholds in the oral cavity, but these have focused on normative values rather than individual differences on the tongue (e.g.^31^), or comparisons between patients and healthy controls on the inner cheek^32^. Thus, one objective of the current research was to measure and quantify differences in pressure point sensitivity on the tongue in nominally healthy adults.

Due to the importance of texture in food products like chocolate and the potential for substantial individual differences in oral touch across the population, there is a need to assess oral tactile function in consumers and determine how this may relate to specific food products. As far as we know, no prior work has reported associations between pressure point sensitivity in the oral cavity in relation to the perception of texture attributes such as particle size in a food product. Accordingly, the aims of the present study were to (a) test the ability of consumers to discriminate between different particle sizes in chocolate around the widely claimed critical value of ~25 μm, (b) to quantify oral pressure point detection and discrimination threshold estimates in healthy consumers, and (c) measure the relationship between oral pressure point threshold estimates with the discrimination of particle size in commercial dark chocolate.

## Materials and Methods

### Study overview and participants

A total of 111 volunteers were recruited for two experiments. An online screening questionnaire was completed by 500+ individuals in State College, Pennsylvania, USA, and the surrounding area: regular chocolate consumers who were not pregnant and did not have food allergies, or any history of facial injury, recent dental work, any known smell or taste defects, or any mouth piercings, were invited to participate. Fifty individuals (13 men, 37 women) completed Experiment 1a, a taste test with experimentally manipulated chocolates produced in our pilot plant (details below). These 50 individuals were later invited back to evaluate a second set of experimental chocolates; 10 newly qualified respondents were also invited, resulting in a total of 60 participants (18 men, 42 women) for Experiment 1b. In a separate study (Experiment 2), oral touch sensitivity was measured one-on-one with a trained experimenter in 51 new participants (10 men, 41 women); no one from Experiment 1a or b participated in Experiment 2. Of the 51 participants who had their oral touch sensitivity quantified in Experiment 2, 44 participants (7 men, 37 women) later returned to our laboratory to evaluate two commercially produced chocolates.

All experiments were performed in accordance with relevant guidelines and regulations for research with human participants. For Experiment 1, all procedures were exempted from full IRB review by the Penn State Office of Research Protections under the consumer testing exemption in 45 CFR 46.101(b)(6). For Experiment 2, the protocol was reviewed and approved by Penn State Institutional Review Board (Protocol #00006365). All participants provided informed consent; implied consent was obtained via a click through question on the computer for Experiment 1, and written consent was provided for Experiment 2. Participants received a small cash incentive for their time, and all testing was conducted in the Erickson Food Science Building at the Pennsylvania State University.

### Production of experimental chocolates for experiment 1a & 1b

For Experiment 1a, West African chocolate liquor (sourced from Cargill Cocoa & Chocolate, Mount Joy, PA) was combined with extra fine granulated cane sugar (Golden Barrel, Honey Brook, PA) in small white plastic buckets (10-inch diameter) and thoroughly mixed. Six batches of liquor and sugar were created and run through a Lehmann model 5.B 3-roll refiner set to different particle sizes. The refiner was set to D_90_ particle size values of approximately 30, 35, 40, 50, 60, and 80 micrometers (microns; μm) and each batch was refined. These values represent a very large range of particle sizes for chocolate, as the majority of commercial products produced fall below 30 μm in D_90_ size^9^. After refining, the refiner flake (sugar + cocoa liquor) for each particle size was conched in a Hobart A200 table top conch with an attached water bath. Each sample was conched for 7.5 hours with the water bath set to 55 °C. At the 7.5-hour point, deodorized cocoa butter and soy lecithin (Bioscience Food Solutions GmbH, Siegburg, Germany) were added and the batch was conched for an additional 30 minutes. The final formulation for all samples was: 48.9% sucrose, 43.7% cocoa liquor, 6.86% cocoa butter and 0.5% soy lecithin (by mass). The final chocolate sample formulations were at a fat content of ~32%. (This level was selected because the fat content of chocolate can affect rheological properties like viscosity; however, above ~30%, the effect of fat on viscosity is negligible^33^). After conching was completed, samples were removed from the conch and were stored in their original buckets until sensory testing. Final particle sizes for the two series of stimuli created with the roll refiner are shown in Table 1.

**Table 1:** Particle sizes (D_90_ in micrometers) for chocolate produced via a 3-roll refiner (Experiment 1a) and a ball mill (Experiment 1b).

	Experiment 1a (Roll Refiner)				Experiment 1b (Ball Mill)			
	Series 4		Series 3		Series 2		Series 1	
Constant Ref	52.4 µm		41.3 µm		29.3 µm		26.4 µm	
			A	32.4 µm			A	19.3 µm
	A	38.3 µm	B	38.3 µm	A	21.9 µm	B	21.9 µm
	B	41.3 µm		—	B	26.5 µm	Blind Ref	26.5 µm
		—	C	52.4 µm	Blind Ref	29.3 µm	C	29.3 µm
	C	51.7 µm	D	51.7 µm	C	33.2 µm	D	33.2 µm
	D	79.3 µm			D	35.7 µm		

Columns represent decreasing D_90_ particle size of the constant reference for that series, from left to right. Within a specific series (column), samples are labeled with letters A-D for convenience of the experimenter; these labels were never seen by participants, as random 3-digit blinding codes were used during sensory testing. Nominal sizes for each batch were based on settings of the equipment during manufacturing; after sensory testing had been completed, quantitative values were determined via laser diffraction.

Within an experiment, 6 batches of chocolate were produced at each nominal size in single batches. Thus, sample A from series 4 and sample B from series 3 are chocolate from the same batch.

Due to limitations of the roll refiner used in Experiment 1a, a Union Process Attritor grinding ball mill was used to produce chocolates with D_90_ particle sizes smaller than 30 μm for Experiment 1b. An attached water bath (set to ~37.8 °C/100 °F) was used to cool the sample during production. As above, chocolate liquor was combined with granulated cane sugar in round buckets and mixed in the same formulation. Six batches of chocolate at different particle sizes were refined using the ball mill. The ball mill was run at 1750 rpm with 5 millimeter (mm) diameter stainless steel balls for differing times to create samples with varying particle sizes. Final particle sizes for each of six batches are shown in Table 1. After grinding in the ball mill, finished chocolate was separated from the stainless-steel balls using a colander and shake table. Samples were then stored until sensory testing.

### Particle size analysis

Particle size analysis of all samples was conducted using a Malvern Mastersizer® 3000 laser diffraction particle size analyzer. Chocolate samples were prepared using a standardized procedure developed in conjunction with the Penn State Materials Characterization Laboratory and Malvern Instruments^34^. Representative output from this protocol can be found in the Supplementary Materials.

### Sensory testing with experimental chocolates for experiment 1

Chocolate samples were melted in a warming box set at ~37.8 °C (100 °F) before testing by naïve chocolate consumers. Samples were evaluated as melted chocolate – this was done to minimize any potential variation due to differences in chocolate eating style (i.e., fast chewers, thorough chewers, and suckers)^35^, and because prior work indicates the perception of particles begins once samples are melted in the mouth^36^. Samples were served to seated participants in two-inch plastic cups containing ~4 g of melted dark chocolate; each cup was labeled with a random 3-digit blinding code. Participants evaluated pairs of chocolate samples for perceived grittiness in a two-alternative forced choice (2-AFC) task using the method of constant stimuli^15^. Here, the term *task* refers to what participants were asked to do (choose the grittier stimulus) while the term *method* refers to the manner in which stimuli were presented to the participant.

In Experiment 1a, participants were asked to evaluate two series of samples, with four pairs in each series (i.e., a total of 8 chocolates). Following the method of constant stimuli, each series included a fixed reference that was compared against 4 experimental samples (two with particle sizes above the reference, and two below) in a pairwise fashion. In Experiment 1b, participants evaluated a total of five pairs for the two series: samples were similar to those in Experiment 1a (two above, and two below the reference), with the addition of a blind control (i.e., the reference against itself). After evaluating each pair, participants were asked to rinse with water, as prior data indicate water is superior to other palate cleaners^37^; a 30-second break was enforced between each pair via software. Presentation order was counterbalanced using a balanced Latin squares design. All data were collected using Compusense Cloud software (Guelph, ONT, Canada). Using Microsoft Excel (Microsoft, Redmond, WA), the sum of the number of total pairs correct were calculated, and the percentage of times a stimulus was judged as “grittier than” the reference was determined for each pair. Just Noticeable Differences and Weber fractions were calculated as described by Camacho and colleagues^14^.

### Assessing oral somatosensory function for experiment 2

Prior to testing, participants provided consent and answered standard demographic questions (e.g., age, gender). They were also asked about their general health and average speech use^38^, health of their lower face^24^, use of tobacco socially or regularly, if they were diabetic or used insulin, and whether they had a neurologic injury like a stroke or concussion. During testing, participants were seated in a private windowless exam room. Tactile detection and discrimination threshold estimates were obtained using a set of calibrated Von Frey Hairs (VFH) ranging from 0.008 g to 15 g (Aesthesio® Precision Tactile Sensory Evaluators, DanMic Global LLC, San Jose CA). Detection and discrimination assessments were completed bilaterally on the right and left edge of the tongue just lateral to the tongue tip as well as at midline (“center”) on the tongue tip. The order of the testing locations was counterbalanced across participants. Participants were allowed to rinse with water at any point, and a break of 3 minutes was enforced between detection and discrimination threshold assessments. All equipment was cleaned immediately using 70% ethyl alcohol prep pads after testing was finished with a participant, and again prior to testing with a new participant. All question and threshold data were collected and recorded on paper ballots. Data was entered into Microsoft Excel software for analysis.

### Detection thresholds using VFH for experiment 2

Tactile detection threshold estimates were determined using a two-alternative forced choice (2-AFC) task^39^. Participants were required to indicate which of the two sequentially presented observation intervals contained the test stimulus; this is sometimes called a temporal 2-AFC or two-interval-forced-choice (2-IFC) task. Participants heard the researcher say “trial one” and “trial two”, with the stimulus being presented randomly in only one trial. Using one or two fingers, the participant indicated in which trial they felt the stimulus; if participants were unsure, they were told to make their best guess (i.e., a forced choice). All participants started at 1.0 g of nominal pressure. Threshold measurement was conducted with a 3 down/1 up staircase method. The amount of force applied from VFH is discrete and the progression is nonlinear, as the nominal pressure levels (in grams) are predetermined by the manufacturer (i.e., 0.002, 0.02, 0.04, 0.07, 0.16, 0.4, 0.6, 1, 1.4, 2, etc). A new set of filaments was purchased prior to testing, and no attempt was made to verify manufacture provided values. When a participant gave three correct detections at a specific stimulus level, the researcher decreased the VFH filament to the next lower level. If the participant was incorrect, the researcher increased the stimulus to the next level up. Final detection threshold estimates were defined as the mean of 5 reversals (‘crossings’) in the staircase. No feedback was given to participants.

### Discrimination thresholds using VFH for experiment 2

Continuing with the Von Frey Hair monofilaments, discrimination threshold estimates were determined using the approach of Etter and colleagues^24^. In contrast to the detection task described above, for discrimination, participants received stimuli in both trials one and two. They were asked to indicate, using one or two fingers, which trial had the “stronger or harder” pressure. The trial with greater force was counterbalanced. As the monofilament stimulators come in fixed levels of force (see above), discrimination trials started at three levels above their detection threshold. For example, if a participant’s detection threshold was 0.02 grams, they started their discrimination thresholds at 0.16 grams. The same criteria for correct and incorrect responses, and stopping points were used as above.

### Discrimination of two commercially available chocolates for experiment 2

Participants received two squares of solid commercially available dark chocolate at ambient temperature in 1-inch diameter cups labeled with random 3-digit blinding codes. The chocolates were served solid at room temperature (in contrast to Experiment 1), both for simple logistics of one-on-one testing, and to further increase the ecological validity of these data. Participants were asked to identify the grittier sample in a 2-AFC task. The two chocolates were Scharffen Berger Bittersweet Dark and ChocoLove Strong Dark, both with 70% cocoa, purchased locally. Participants rinsed with water before tasting and were allowed to rinse at any other point during tasting. Testing ended after the chocolate tasting was complete.

### Data analysis

Pressure-point detection and discrimination threshold estimates were calculated for the right, center and left tongue tip. Significant differences in participant detection and discrimination threshold estimates by location (Right, Center & Left) were determined using paired t-tests. Linear regression was used to test for a possible relationship between a participant’s detection and discrimination threshold estimate. No subgroup analyses on the basis of gender were performed: we did not have any such a priori hypotheses, and the unequal gender split in our participants would make any such analysis underpowered and potentially misleading.

Because the discrimination thresholds were clearly bimodal (see below), participants were dichotomized into high and low sensitivity groups using the antimodal value (0.6 g) as the cutoff. Potential relationships between the groups were assessed via chi-square analysis. Similarly, relationships between chocolate discrimination ability and oral touch phenotype (high/low groups for point pressure) were assessed via chi-square tests.

As D_90_ and D_(4,3)_ represent different summary parameters drawn from the same particle size distribution function for an individual batch of chocolate, the results below focus on the D_90_ values both for simplicity and clarity, and because these values have greater industrial relevance. Interested readers can find D_(4,3)_ data in the Supplemental Materials.

## Results

### Just noticeable differences and weber fractions

**Figure 2:**
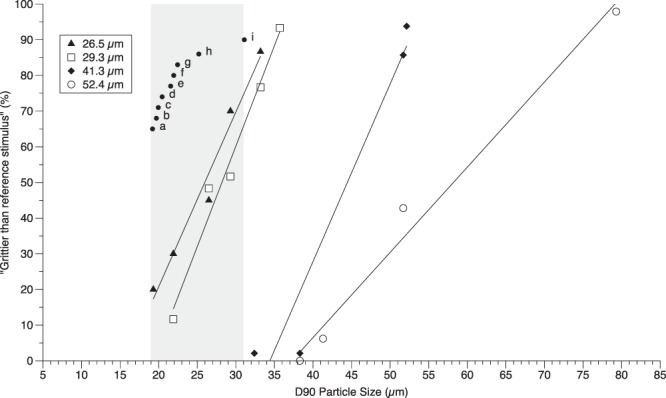
Proportion of times each experimentally produced chocolate was judged “grittier than the reference stimulus”. The D_90_ particle size of the reference for each series is shown in the legend. For context, the gray shaded box indicates the range of particle size for commercially available dark chocolates analyzed via laser diffraction. The commercial products were: (a) Lindt Dark; (b) ChocoLove Strong Dark; (c) Wegmans Supermarkets Dark; (d) Ghirardelli Dark 72%; (e) Cacao Godiva Dark; (f) Hershey’s Special Dark; (g) Ghirardelli Dark 60% Cacao; (h) Scharffen Berger Bittersweet Dark; (i) Cavalier Dark.

For experiments 1a and 1b, the D_90_ particle sizes for the experimentally produced chocolates are shown above in Table 1. Figure 2 shows the percentage of times each chocolate was identified as being grittier than the constant reference. For additional context, dots labeled with lower case letters are also shown: these represent particle size values from commercially available chocolates using the same measurement procedure. These commercial products were not evaluated by participants in Experiment 1 and are shown for context. The D_90_ particle size of commercial samples ranged from 19.2 µm to 31.1 µm, which largely overlaps with the range of values obtained for the experimentally produced chocolates in series 4 and 3. Linear fits were plotted for each series, and were used to calculate the JND values and corresponding Weber Fractions (K).

For three smaller series (i.e., those with references of 26.5 µm, 29.3 µm and 41.3 µm D_90_ values), the JND values were relatively constant, around ~5 µm. Under Weber’s Law, the JND should be proportional to the size of the stimulus, so unsurprisingly, the JND for the largest series (i.e., a reference stimulus of 52.4 µm) was substantially larger than the other series, with a JND value of 10.5 µm. However, given this, we would have also expected the JND for Series 3 to be slightly greater; the reason for this small discrepancy is unknown. Table 2 details the values for the JND and reference D_90_ particle size for each series. The empirically determined Weber Fractions (K) for series 4, 3, 2 and 1 were 0.20, 0.12, 0.15, and 0.19, respectively, with an overall mean of 0.17. When D_(4,3)_ particle sizes were used to calculate JND’s (see Table S2 in Supplemental Materials), the JNDs increased as the particle size of the reference stimulus increased, and the Weber Fraction ranged from 0.07 to 0.14 (see Table S3).

**Table 2:** Estimates of the Just Noticeable Differences (JNDs) empirically determined for the 4 sets of stimuli shown in Fig. 2.

Series	D_90_ particle size of reference	JND D_90_
Series 1	26.5 µm	5.04 µm
Series 2	29.3 µm	4.44 µm
Series 3	41.3 µm	5.02 µm
Series 4	52.4 µm	10.5 µm

### Pressure point sensitivity via VFH in experiment 2

Pressure point detection threshold estimates were obtained for the left and right anterior lateral edge and midline (“center”) of the tongue tip in 51 participants. Pressure point discrimination thresholds were only obtained from 47 of these participants due to a testing error.

**Figure 3:**
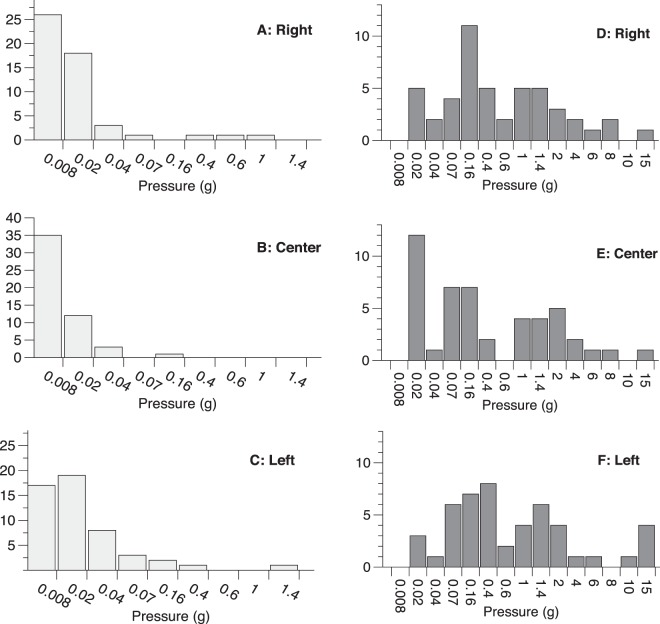
Measures of oral somatosensory function collected from young, nominally healthy adults in Experiment 2. In each panel, the x-axis is the nominal pressure applied, and the y-axis is the number of participants. Detection threshold estimates are shown on the left-hand column for the anterior lateral edge of the tongue on the participant’s right (top row), midline center of the tongue tip (middle row) and the anterior lateral edge of the tongue on the participant’s left (bottom row). Discrimination threshold estimates are shown in the right-hand column, at the same locations. The detection thresholds are from 51 participants while the discrimination thresholds are from 47 of the same individuals.

Threshold estimates for detection and discrimination at each tongue location are shown in Fig. 3 and Table 3. The detection threshold estimates showed evidence of a floor effect, as most participants were able to detect stimuli at or below the second lowest stimulus level (0.02 grams of pressure) in the 2-IFC task (i.e., when a stimulus was only presented in one of two temporal intervals). Across tongue regions, the right and left lateral edge of the tongue were not significantly different for detection (p’s > 0.10), but there was some weak evidence that values on the midline of the tongue tip might be different than the left (p = 0.07) and the right edges of the tongue (p = 0.11). Within the same participants, the variance in participants’ discrimination threshold estimates were significantly higher than for detection thresholds at all three tongue loci (F-test; all p’s < 0.004). For the discrimination threshold estimates, there was no evidence of significant differences between the three loci (all p’s > 0.10).

**Table 3:** Summary statistics of the detection and discrimination threshold estimates shown in Fig. 3.

Location	Detection (g)		Discrimination (g)	
	M (SE)	Range	M (SE)	Range
Tongue, right	0.016 (0.023)	0.008–1.0	0.36 (0.052)	0.02–15.0
Tongue, center	0.011 (0.0031)	0.008–0.16	0.22 (0.031)	0.02–15.0
Tongue, left	0.022 (0.028)	0.008–1.4	0.49 (0.072)	0.02–15.0

Values shown are geometric means, standard errors and range.

Although minimal variation was observed for pressure point detection threshold estimates, there was greater variation observed in the ability to discriminate between two different levels of force. Based on the antimodal values apparent in Fig. 3, we split participants into high (PPHigh) and low (PPLow) sensitivity groups for each tongue location tested. Mean values for the groups are shown in Table 4, and as would be expected following classification, the group means were significantly lower for the high point pressure sensitivity group (PPHigh) than the low point pressure sensitivity group (PPLow) at all three loci (all p’s < 0.005).

**Table 4:** Geometric means of discrimination threshold estimates for the more sensitive (PPHigh) and less sensitive (PPLow) groups after dichotomization.

Location	Discrimination (g)	
	PPHigh	PPLow
Tongue, right	0.0756	1.450
Tongue, center	0.0321	0.957
Tongue, left	0.126	2.390

### Texture discrimination in commercial chocolates

After all one-on-one somatosensory testing was complete, participants evaluated two commercial chocolates in a 2-AFC task. Across all participants, two-thirds were able to discriminate between the two commercial chocolates. They systematically identified the Scharffen Berger sample as being ‘more gritty’ than the Chocolove sample: 44 of 67 responses (p = 0.0093, one tail). This is consistent with laser diffraction analysis, as the Scharffen Berger had a D_90_ particle size of 25.20 μm versus 19.70 μm for the Chocolove sample. The significant difference between samples was anticipated, as the difference in particle sizes between the two commercial samples was greater than the JND estimates determined in Experiment 1.

Possible relationships between point pressure discrimination thresholds and discrimination of commercial chocolates were tested; we did not test similar relationships with the detection threshold estimates, given the minimal variation observed in this young, nominally healthy cohort. As above, participants were grouped into PPLow and PPHigh groups for pressure point sensitivity for the right, center and left tongue. In chi-square analysis, there was no significant relationship between pressure point discrimination ability on the right and left lateral edges of the tongue and perception of grittiness in the commercial dark chocolates tested (p’s > 0.20). In contrast, there was evidence of a relationship between pressure point discrimination ability at the midline of the tongue tip, and perception of particles in chocolate (chi-square = 0.0246). That is, 85% (17/20) of the individuals in the PPHigh group correctly identified the chocolate with the larger particle size as being grittier, while the individuals in the PPLow group performed exactly at the level expected by chance (50%; 12 of 24 correct).

## Discussion

Here, we describe two novel experiments on the ability of naïve chocolate consumers to discriminate between different particle sizes in dark chocolate. Individual differences in oral point-pressure sensitivity were assessed as measures of oral somatosensory function, and a significant relationship was observed between differences in clinical measures of oral somatosensory function at the tongue tip and texture perception of dark chocolate. These data advance our understanding of texture perception in the oral cavity, by suggesting that, as with smell and taste, phenotypic differences in oral somatosensory can generalize to real foods.

For experimentally produced dark chocolates with D_90_ particle size from 19 to 33 µm and 22 to 36 µm respectively – that is, in a range comparable of commercially available dark chocolates – the Just Noticeable Differences (JNDs) were 5.04 and 4.44 µm. This suggests that naïve chocolate consumers (i.e., non-experts) are able to perceive differences in chocolate particle size when they differ by only ~5 micrometers (microns). The JND was greater when the size of the reference increased, as would be expected from Weber’s Law. While the larger stimuli with correspondingly bigger JNDs were above the range normally found in chocolates intended for eating, these data still have some industrial relevance. This is because much of the chocolate produced commercially is intended for use in products such as cookies, biscuits, caramel or nuts, and these ‘inclusions’ are frequently manufactured with larger particle sizes. Indeed, the grittiness of the chocolate is partially masked by the texture of the food itself^12^. Present findings may also help reduce energy use in the chocolate industry (reducing both environmental impact and production cost), as chocolates with smaller D_90_ particle sizes take longer to refine and require substantially more energy in manufacturing^9^. These data also speak to quality control efforts vis-à-vis critical limits: as a matter of definition, variation below the JND will presumably not be noticed by consumers.

Calculations of the Weber Fractions (K) for the four series of melted stimuli in Experiment 1 were roughly constant, with a mean of 0.17 for the D_90_ particle size. As would be expected, the JND for larger particle sizes was larger, similar to many other studies in food and non-food products^14,40,41^. The Weber Fractions determined here in melted chocolate for particle size perception are comparable to those reported for various taste qualities such as sweetness (K = 0.13), saltiness (K = 0.15) and sourness (K = 0.22)^41^. In contrast, chemesthetic stimuli (like the perception of pungency from chilies) show much higher Weber Fractions (K = 0.59–0.64)^40^ than those observed here. Although the psychophysics of particle size perception have not been formally tested previously, present data are also aligned with Weber Fractions reported previously for other textural properties, including viscosity of model beverages (K = 0.26)^14^, creaminess of added fat in beverages (K = 0.16)^42^ and the firmness of edible fats (K = 0.20)^43^. Given that values reported previously for textural attributes like creaminess perception and firmness are similar to those observed here for particle size in chocolate, this suggesting that there may be shared mechanisms for oral texture perception in humans.

Regarding individual differences in oral somatosensory function on the tongue, minimal variation was observed in pressure point detection, regardless of locus, as their appears to be a floor effect, as the lightest Von-Frey Hairs (VFHs) were clearly perceptible to young nominally healthy adults without oral pathology. Similar effects have been reported in other work on healthy young adults, regardless of whether thresholds were determined with an adaptive staircase (like the one used here)^24^ or the method of limits with ascending and descending series^31^. Despite the minimal variation seen across participants, there was also some evidence here that detection thresholds may be lower on the midline of the tongue as compared to the lateral edge on the right and left near the tip. Thus, present data bring new empirical support to the idea that the tip of the tongue is the more sensitive than the sides^44^. We believe this is a novel finding: prior work with VFH suggests the tongue tip is more sensitive than the face (right cheek) or upper gums^31^, but we cannot find any prior data comparing tongue regions. Increased sensitivity at tongue midline may be due, in part, to the fungiform papillae (FP) present on the tongue tip, as other work suggests those with higher FP density have higher lingual tactile sensitivity in a letter recognition task^45^. Yackinous reported that those who perceived more bitterness from 6-n-propylthiouracil (i.e., so-called supertasters) were more sensitive to point-pressure stimulation with VFH on the center tongue^29^, and that supertasters had higher fungiform papillae counts on the anterior tongue^46^. However, those studies did not directly assess potential relationships between pressure point sensitivity and FP density, and we did not collect data on FP density here, so additional work is needed to explore possible relationships between papillae density and oral somatosensory function.

In contrast to the detection threshold data, substantially more variability was observed in discrimination threshold estimates across individuals. We did not find evidence of any significant differences in terms of discrimination ability across loci; however, discrimination threshold estimates did seem to show the same pattern as the detection thresholds, with the tongue tip having slightly lower threshold estimates. Additional work seems warranted. In direct comparison with other recent data^24^, the discrimination threshold estimates for the right and left lateral edge of the tongue were slightly higher here; the reason for this is unknown. Conversely, discrimination threshold estimates for pressure point sensitivity for the tongue tip have not been reported previously, so we are unable to make any comparisons.

Critically, the ability to detect a stimulus and to discriminate between stimuli are subtly different cognitive tasks. However, for oral touch, it has not been reported whether these might covary within an individual – i.e. does high detection ability correspond with high discrimination ability? Here, no relationship was observed between a participant’s detection and discrimination ability for pressure point sensitivity, with the caveat that a potential relationship cannot be ruled out entirely, given the restricted range of responses for the detection thresholds in this nominally healthy cohort. Alternatively, the apparent lack of a relationship may also be due to the fact that detection and discrimination procedures are different cognitive tasks^39^. In a detection threshold, the stimulus must be stronger than some critical amount in order to be consciously observed^47^, whereas in a discrimination threshold, participants are required to distinguish differences in intensity between two stimuli.

In experiment 2, we asked participants to evaluate two commercially available dark chocolates that differed in particle size, and critically, this difference was just above the JND determined with experimentally produced chocolates in Experiment 1. Indeed, the two commercial chocolates were roughly the same particle sizes (19 μm and 26 μm) as two of the experimentally produced chocolates (samples A and the blind reference from Series 1): this allows for a direct comparison across the two experiments. In both, the groups were able to discriminate between the samples (80% correct, and 66% correct, respectively). The slightly better performance for the experimental chocolates may be explained by the slightly larger difference in particle size (7.2 um versus 5.5 um for the pair of commercial chocolates, although both were above the estimated JND of ~5 micrometers (microns).

Limitations of this work should be noted. Chocolate is a convenient model food that is both self-stable and highly acceptable to participants, as well as being amenable to experimental manipulation in terms of particle size. However, it is somewhat unique in that most foods are not a semi-solid suspensions of fine particles in a continuous fat phase. As such, present findings may not generalize to other foods, or to textural properties beyond grittiness. For example, in a recent pilot study, we failed to find any evidence to support a relationship between differences in point pressure sensitivity and viscosity discrimination in maltodextrin solutions (Alcala, Etter, & Hayes, unpublished data). Also, sample presentation differed between Experiments 1 and 2 (melted versus solid chocolate). Melted chocolate was used in the JND experiment to minimize possible effects of eating styles (see^35^). Whether such differences could modify the relationships seen in Experiment 2 remains unknown; while unlikely, additional work would be needed to formally test this. Last, we did not test for any sex or gender effects here. While sex effects are common in taste research (e.g.^48,49^), touch research on non-oral surfaces either fails to find sex effects on the face^28^ or tongue^31^, or suggests that apparent sex differences may be artifacts due to size differences^50^. Additional work on the role of sex and oral cavity size is needed.

Here, we tested whether there was a relationship between oral touch sensitivity and the perception of particle size. In Experiment 2, there were two distinct groups of participants in terms of discrimination thresholds, and they showed substantial variation in their ability to discriminate between different amounts of force applied to the tongue. When participants were split into groups based on pressure point sensitivity – high (PPHigh) and low (PPLow) acuity – there was a significant relationship between chocolate discrimination and pressure point sensitivity for the PPHigh group on the center tongue. However, a similar relationship was not seen for data from the lateral edge of the tongue. These findings are novel, as we are unaware of previous work showing a relationship between oral pressure sensitivity and ability to detect differences in particle size in a food product. Collectively, these findings suggest that texture detection mechanisms which underpin point pressure sensitivity likely contribute to the detection of particle size in food like dark chocolate.

## Supplementary information

Supplementary Information
