# Supplementary material for: Oral somatosensatory acuity is related to particle size perception in chocolate
Source: Sci Rep. 2019 May 15;9:7437. doi: 10.1038/s41598-019-43944-7 (PMC6520395; doi:10.1038/s41598-019-43944-7)
Supplement: Supplementary file 1 — Supplementary Information [file 41598_2019_43944_MOESM1_ESM.docx]

Supplementary Information for:

**Oral somatosensatory acuity is related to particle size perception in chocolate**

Scott P. Breen ^1,2^, Nicole M. Etter^3^, Gregory R. Ziegler^2^, John E. Hayes ^1,2,*^

Sensory Evaluation Center, and Department of Food Science, The Pennsylvania State University

^1^Sensory Evaluation Center and ^2^Department of Food Science, College of Agricultural Sciences

^3^Department of Communication Sciences and Disorders, College of Health and Human Development

The Pennsylvania State University, University Park, PA 16802

*Corresponding Author:

Dr. John E. Hayes

Department of Food Science

Pennsylvania State University

220 Food Science Building

University Park, PA 16802

814-863-7129 (voice)

[jeh40@psu.edu](mailto:jeh40@psu.edu)

The particle size distributions (PSDs) for chocolate samples produced using the via ball mill (i.e., smaller particle sizes for Series 2 and 1) were more unimodal than the PSDs for chocolate samples produced via the roll refiner (i.e., larger particle sizes for Series 3 and 4), which were more bimodal in shape. This was not unexpected given differences in how the two refining methods function.

**Figure S1. Example PSD for Chocolate C from *series 3* in Table 1.**


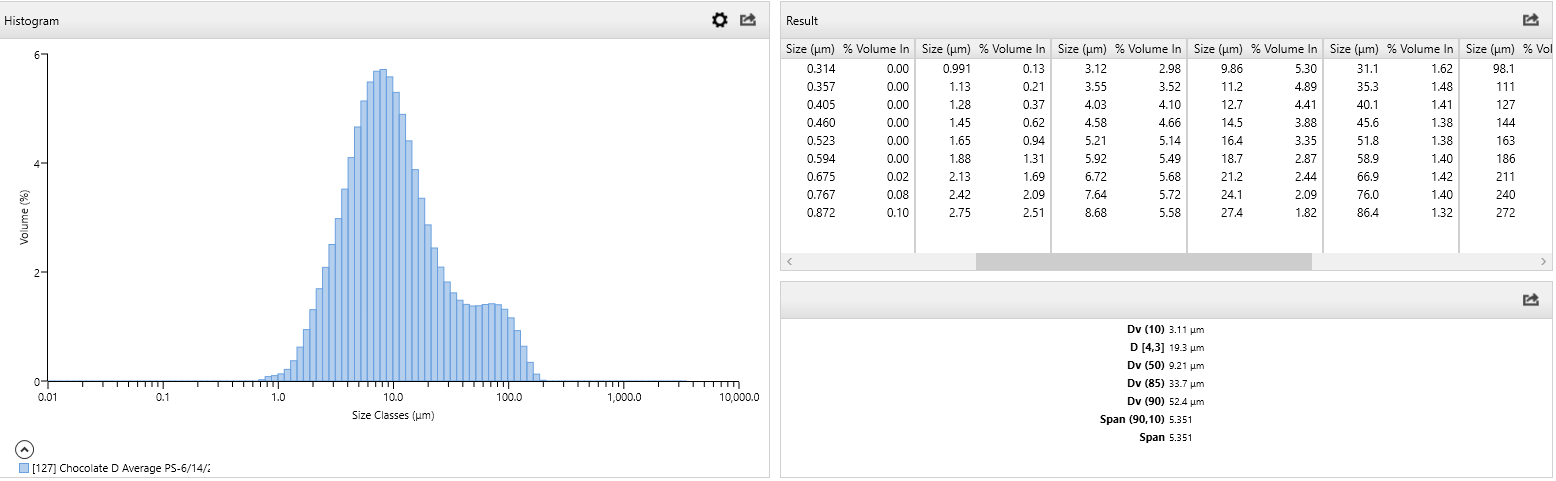


**Figure S2. Example PSD for Chocolate C from *series 1* in Table 1.**


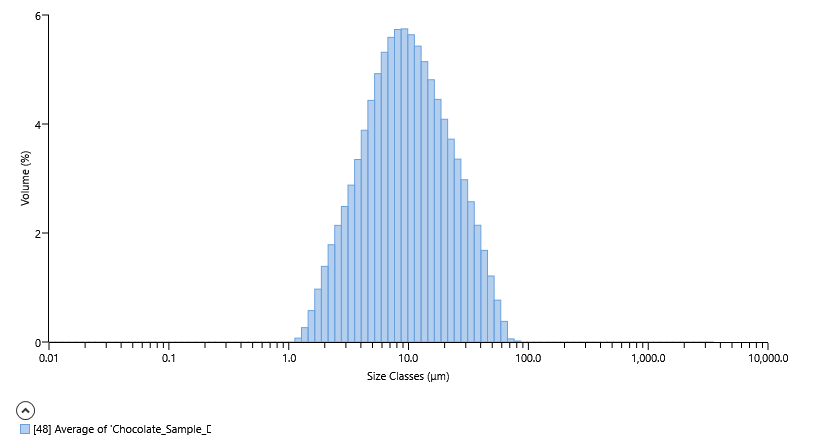


Any potential differences in the shapes of the PSDs are minimized by focusing on the D_90_ results reported in the main paper, as this also happens to be the more industrially relevant parameter. Thus, we do not believe potential influences of PSD share meaningfully influence the conclusions reported in our paper.

For completeness, the D_4,3_ values are provided below, with appropriate caveats about the shape of the PSD.

#### Table S1. Particle sizes (D_4,3_ in microns) for chocolate produced via a 3-roll refiner (Experiment 1a) and a ball mill (Experiment 1b). Columns represent decreasing D_90_ particle size of the constant reference for that series, from left to right. Within a specific series, samples are labeled with letters A-D for convenience of the experimenter; these labels were never seen by participants, as random 3-digit blinding codes were used during sensory testing. Nominal sizes for each batch were based on settings of the equipment during manufacturing; quantitative values were determined via laser diffraction after sensory testing had been completed.

|  | Experiment 1a  (Roll Refiner) | | | |  | Experiment 1b  (Ball Mill) | | | |
| --- | --- | --- | --- | --- | --- | --- | --- | --- | --- |
|  | Series 4 | | Series 3 | |  | Series 2 | | Series 1 | |
| Constant Ref | 19.3 µm | | 18.4 µm | |  | 9.6 µm | | 8.9 µm | |
|  |  |  |  |  |  |  |  |  |  |
|  |  |  | A | 13 µm |  |  |  | A | 7.6 µm |
|  | A | 15.2 µm | B | 15.2 µm |  | A | 8.1 µm | B | 8.1 µm |
|  | B | 18.4 µm |  | – |  | B | 8.9 µm | Blind Ref | 8.9 µm |
|  |  | – | C | 19.3 µm |  | Blind Ref | 9.6 µm | C | 9.6 µm |
|  | C | 18.4 µm | D | 18.4 µm |  | C | 9.8 µm | D | 9.8 µm |
|  | D | 26 µm |  |  |  | D | 10.2 µm |  |  |
| Within an experiment, 6 batches of chocolate were produced at each nominal size in single batches. Thus, sample A from series 4 and sample B from series 3 are chocolate from the same batch. | | | | | | | | | |

Table S2. Estimates of the Just Noticeable Differences (JNDs) empirically determined for the 4 sets of chocolates using the D_(4,3)_ particle sizes.

| Stimulus Series | D_(4,3)_ particle size of reference | JND D_(4,3)_ |
| --- | --- | --- |
| Series 1 | 8.9 µm | 0.876 µm |
| Series 2 | 9.63 µm | 0.700 µm |
| Series 3 | 18.4 µm | 1.54 µm |
| Series 4 | 19.3 µm | 2.71 µm |

#### Table S3. Weber fractions (k) using the D_(4,3)_ particle sizes for the 4 series of chocolates tested in Experiment 1.

|  | D_(4,3)_ |
| --- | --- |
| Series 1 | 0.09 |
| Series 2 | 0.07 |
| Series 3 | 0.08 |
| Series 4 | 0.14 |
| Average | 0.10 |
